# Mandibular asymmetry: A proposal of radiographic analysis with public domain software

**DOI:** 10.1590/2176-9451.19.3.052-058.oar

**Published:** 2014

**Authors:** Alexandre Durval Lemos, Cintia Regina Tornisiello Katz, Mônica Vilela Heimer, Aronita Rosenblatt

**Affiliations:** 1 Professor, Department of Dentistry, State University of Paraíba, UEPB.; 2 Assistant professor, Department of Social Dentistry, Federal University of Pernambuco, UFPE.; 3 Full professor, Department of Social Dentistry, University of Pernambuco, UPE.

**Keywords:** Panoramic radiography, Imaging diagnosis, Mandible

## Abstract

**Objective:**

This preliminary study aimed to propose a new analysis of digital panoramic radiographs for a differential diagnosis between functional and morphological mandibular asymmetry in children with and without unilateral posterior crossbite.

**Methods:**

Analysis is based on linear and angular measurements taken from nine anatomic points, demarcated in sequence directly on digital images. A specific plug-in was developed as part of a larger public domain image processing software (ImageJ) to automate and facilitate measurements. Since panoramic radiographs are typically subject to magnification differences between the right and left sides, horizontal linear measurements were adjusted for greater accuracy in both sides by means of a Distortion Factor (DF). In order to provide a preliminary assessment of proposed analysis and the developed plug-in, radiographs of ten patients (5 with unilateral posterior crossbite and 5 with normal occlusion) were analyzed.

**Results:**

Considerable divergence was found between the right and left sides in the measurements of mandibular length and position of condyles in patients with unilateral posterior crossbite in comparison to individuals with normal occlusion.

**Conclusion:**

Although there are more effective and accurate diagnostic methods, panoramic radiography is still widespread, especially in emerging countries. This study presented initial evidence that the proposed analysis can be an important resource for planning early orthodontic intervention and, thus, avoid progression of asymmetries and their consequences.

## Abstract (pt)

**Objetivo:**

esse estudo preliminar teve como objetivo propor uma nova análise de radiografias panorâmicas digitais para o diagnóstico diferencial entre a assimetria mandibular funcional e morfológica em crianças com e sem mordida cruzada unilateral posterior.

**Métodos:**

a análise se baseia em medições lineares e angulares, tomadas a partir de nove pontos anatômicos, demarcados em sequência, diretamente nas imagens digitais. Um *plugin* específico foi desenvolvido para automatizar e facilitar as medições, como parte integrante de um *software* de processamento de imagem de domínio público (ImageJ). Para maior acurácia, uma vez que as radiografias panorâmicas são sujeitas a distorções entre os lados direito e esquerdo, as medidas lineares horizontais foram ajustadas em ambos os lados, utilizando-se o Fator de Distorção (DF). A fim de fornecer uma avaliação preliminar da análise proposta e do *plugin* desenvolvido, radiografias de 10 pacientes (5 com mordida cruzada unilateral posterior e 5 com oclusão normal) foram analisadas.

**Resultados:**

divergência considerável foi encontrada entre os lados direito e esquerdo nas medições do comprimento do corpo da mandíbula, bem como a posição dos côndilos, em pacientes com mordida cruzada posterior, em comparação a indivíduos com oclusão normal.

**Conclusão:**

apesar de existirem métodos mais eficazes e precisos de diagnóstico, a radiografia panorâmica ainda é muito difundida, especialmente em países emergentes. Esse estudo apresentou evidência preliminar de que a análise proposta pode ser um recurso importante para o planejamento de intervenção ortodôntica precoce, de modo a evitar progressão das assimetrias e suas consequências.

## INTRODUCTION

Advances in medical and biological sciences in recent years and the growing importance of determining the relationship between structure and function have made imaging analysis an increasingly important discipline.^1^ Healthcare professionals, especially dentists, depend on analyses from radiology centers; however, the software programs designed for this purpose are expensive and restricted to the services of these centers. Thus, the use and disclosure of an easy-to-use public domain program for analysis of digital images is of paramount importance.

ImageJ occupies a unique position as a public domain software (www.rsb.info.nih.gov/ij/) that can run on any operating system (Macintosh, Windows, Linux and even a PDA operating system). This software is easy to use, can perform a full set of imaging manipulations and has a huge and knowledgeable user community.^1^ Wayne Rasband is the core author of ImageJ. Its first release (version 0.50) was on September 23^rd^, 1997 and its most recent version (1.47h) was released on December 23^rd^, 2012. After developing the Macintosh-based image bank for the National Institutes of Health (NIH) during 10 years, Rasband made the brave decision to start afresh with ImageJ using the Java programming language (the letter J in the name stands for Java), which freed the software from an individual operating system.^2^ According to the NIH, the software has been downloaded from its web site tens of thousands of times, with a current rate of about 24,000 downloads per month.

ImageJ incorporates a number of useful tools for digital image processing, including determination of linear and angular measurements, calculation of areas, particle analysis, cell counts, etc. This tool has been employed in Medicine (with more than 200 published researches) as well as in other fields of knowledge, such as Engineering, Physics, Astronomy, Computer Science and Chemistry. However, few studies involving the use of ImageJ in the field of Dentistry have been published.^2,3,4^ A search of Pubmed, EBSCO and Scopus databases using the keywords Dentistry and ImageJ revealed 39 studies, only three of which were in the field of Orthodontics.

Studies suggest that patients with unilateral posterior crossbite often exhibit mandibular asymmetry stemming from a functional deviation of the mandible.^5-8^ Routine screening procedures for dental and craniofacial disorders and bilateral examinations of the stomatognathic system are needed. Since panoramic radiographs provide this information, such images could be used as a routine tool for diagnosis and treatment planning. Panoramic radiographs have been used to assess right and left height differences in the condyle, ramus and total mandible height for the definition of asymmetries.^9-12^

Radiographic analyses found in the literature are restricted to the diagnosis of morphological asymmetry in the mandible.^7,8,13,14,15^ Thus, the aim of the present study was to propose a new analysis of panoramic radiographs for a differential diagnosis between functional and morphological asymmetry in children with and without unilateral posterior crossbite using the ImageJ software.

## MATERIAL AND METHODS

The aim of the present preliminary study on differential diagnosis between morphological and functional mandibular asymmetry was to propose a new analysis method involving the use of a public domain software. To this end, digital radiographs from ten patients were analyzed - five with unilateral posterior crossbite and five with normal occlusion. Patients' average age was nine years old. The present study was performed in the city of Campina Grande, in the state of Paraíba, in the northeast of Brazil. It was approved by Paraíba State University Institutional Review board (CAAE: 3201.0.000.133-10).

The criteria for patients with normal occlusion were as follows: Class I canine and molar relationships with minor or no crowding, normal growth and development and well-aligned maxillary and mandibular dental arches; presence of all teeth except for third molars; good facial symmetry (clinically determined); no significant medical history; no functional deviation of the mandible; and no history of trauma or previous orthodontic treatment.

The criteria for patients with posterior crossbite were as follows: unilateral posterior crossbite with at least two posterior teeth in crossbite; mandibular dental midline deviation of at least 1 mm to the crossbite side; functional deviation of the mandible; no systemic disease and no developmental or acquired craniofacial or neuromuscular deformities; no remarkable facial or occlusal asymmetry; no history of orthodontic treatment; no missing teeth (excluding third molars); and no extensive carious lesions or pathologic periodontal condition.

Images were taken with a digital Orthophos DS panoramic radiograph machine (Sirona Dental Systems, Germany) previously standardized (62 Kv, 8 mA and 14.1s exposure time). All radiographs were standardized and taken by the same operator. Patients were positioned with the lips in resting position and the head oriented to Frankfurt horizontal plane.^16^

Anatomical points were marked directly on the digital images using ImageJ software. Based on the objectives of the study, the following landmarks were used: 1- right pterygomaxillary fossa (RPF); 2- anterior nasal spine (ANS); 3- left pterygomaxillary fossa (LPF); 4- most cranial point of left condyle (LHC);^14^ 5- left gonion (LGo); 6- most cranial point of right condyle (RHC);^14^ 7- right gonion (RGo); 8- Pogonion (Pg - midpoint of mandible often seen in panoramic radiographs as a white spot on the midline);^14^ 9- inter-incisive point (IP).

A plug-in was created to automate and facilitate measurement-taking (download and instructions - http://rsbweb.nih.gov/ij/plug-ins/lemos-asymmetry-analysis/index.html).

The following linear (mm) and angular (degree) measurements were taken on both sides of each digital panoramic radiograph:

## Linear measurements

Morphological variables:

- Ramus height (RH): distance between the most cranial point of the condyle (points 4 and 6, as described by Deleurant et al^14^) and the gonion (points 5 and 7).
- Corpus length (CL): distance between gonion (Go) and pogonion (Pg); the gonion was defined as a random midpoint on the posterior curvature of the mandible (intersection points between corpus and ramus).

Functional variables:

- Pg-MSP: distance between pogonion and median sagittal plane, represented by a horizontal link connecting the Pg to the plane.
- IP-MSP: distance between IP and MSP, represented by a horizontal line connecting the IP to the plane.
- CHD: difference between the heights of the right and left condyle (beginning with most superior to the most inferior position; represented by a horizontal line automatically drawn from the CH point of the taller condyle, proceeding to the contralateral side for better visualization in relation to the opposing condyle.

Pg-MSP, IP-MSP and CHD variables were considered as functional, once the panoramic images were taken with subjects in protrusion position; in other words, in functional movement.

## Angular measurement

- Gonial Angle (GA): formed between RH and CL on both sides; results expressed in degrees.

**Figure 1:**
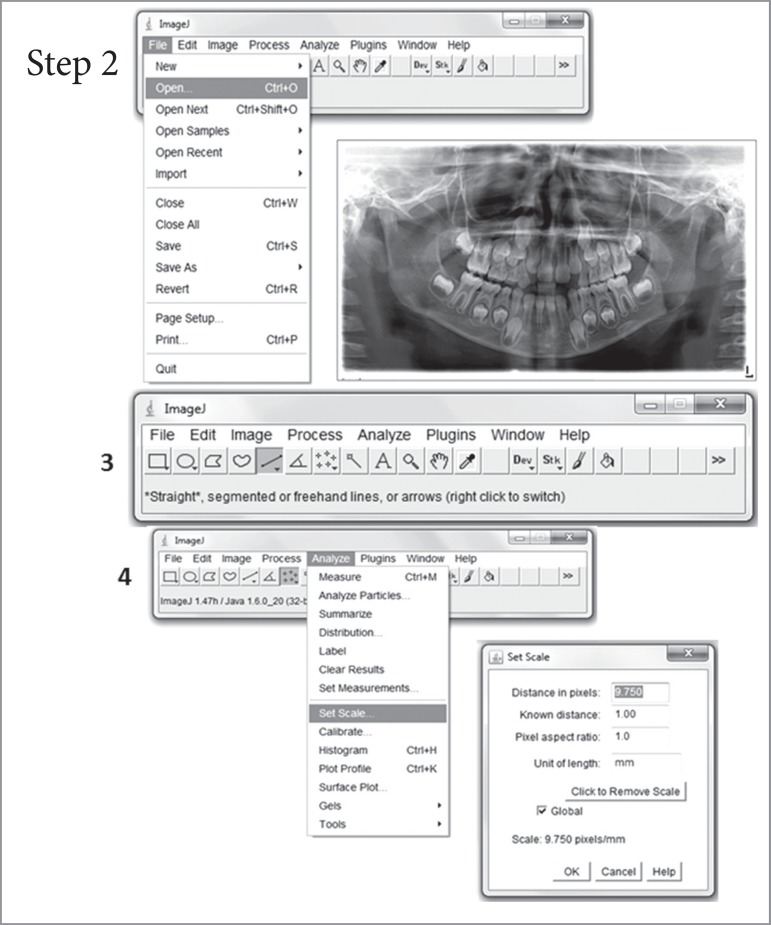
Lemos asymmetry analysis performed on patient with normal occlusion.

**Figure 2:**
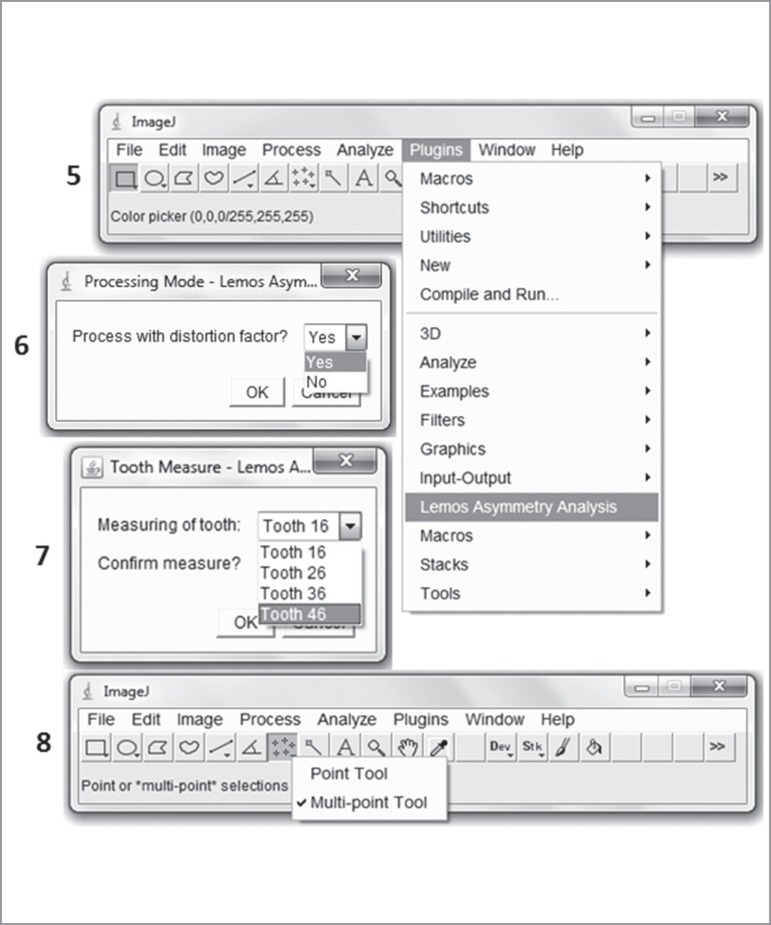
Lemos asymmetry analysis performed on patient with unilateral (right side) posterior crossbite.

After marking the points and determining lines and planes, angular and linear measurements were analyzed (Figs 1 and 2).

Assessment of mandibular asymmetry was performed using the criteria described by Ramirez-Yanes et al,^15^ and categorized as follows: non-significant asymmetry (difference of 0 to 2 mm between the sides of the mandible); light asymmetry (difference of 2 to 3 mm); moderate asymmetry (difference of 3 to 5 mm); and severe asymmetry (difference > 5 mm).

## RESULTS

Tables 1, 2 and 3 display patients' measurements assessed by means of the proposed analysis method. Figures 1 and 2 show the radiographs with the analyses preformed. Considerable divergence was found between the sides in the crossbite group in relation to the corpus length measurement (CL), and positioning of the condyles (CHD) in patients with posterior crossbite in comparison to patients with normal occlusion (Tables 1, 2, 3).

**Table 1:** Statistics for the following variables: ramus height + condyle, corpus length and gonial angle according to side and differences in the crossbite group.

Variable	Side	
	Crossed	Non-crossed
	Mean ± SD	Mean ± SD
Ramus height + condyle	51.9 ± 6.6	51.7± 5.2
Corpus length	68.4 ± 6.2	69.9 ± 7.3
Gonial angle	112.6 ± 6.4	112.8 ± 6.3

**Table 2:** Statistics for the following variables: ramus height + condyle, corpus length and gonial angle according to side and differences in the normal occlusion group.

Variable	Side	
	Right	Left
	Mean ± SD	Mean ± SD
Ramus height + condyle	50.9 ± 1.9	51.3 ± 1.9
Corpus length	75.5 ± 4.7	75.3 ± 4.1
Gonial angle	109.4 ± 3.1	110.0 ± 2.8

**Table 3:** Statistics for the following variables: Pog-MSP, IP-MSP, CHD according to groups.

Variable	Group	
	Experimental	Control
	Mean ± SD	Mean ± SD
Pog-MSP	2.8 ± 2.2	0.8 ± 1.3
IP-MSP	2.0 ± 1.9	0.9 ± 0.9
CHD	3.2 ± 1.7	1.2 ± 0.7

## DISCUSSION

Although it is not considered a public health problem, posterior crossbite stands out as one of the most frequently studied malocclusions in the primary dentition and onset of mixed dentition. Once occurring in the early stages of dental development, self-correction does not generally occur.^18,19^ Early diagnosis and orthodontic intervention allow adequate guidance of maxillary and mandibular growth as well as establishment of an adequate, stable functional pattern in the entire associated musculature, in addition to harmonious development of occlusion.^6,20^ If treatment is not instituted early enough, skeletal remodeling of the temporomandibular joint can occur, thereby leading to permanent deviation from the lower midline and facial asymmetry.^21^ Thus, late treatment is normally more complex, expensive and time-consuming and may involve auxiliary surgical procedures.^22,23^

The present study sought to demonstrate the viability of using ImageJ software as a tool to diagnose mandibular asymmetry in patients with posterior crossbite. Moreover, a plug-in was created to facilitate analysis. This plug-in is a diagnostic tool that can be extended and improved at any time.

Habets et al^9^ proposed one of the first analyses for assessment of mandibular asymmetry developed on a sample of patients with temporomandibular joint problems. Other authors adopted this analysis to measure mandibular asymmetry in patients with posterior crossbite.^7,8^ Although widely employed due to its ease of use, this analysis is restricted to the assessment of vertical measurements (height of corpus and condyle of the mandible) and does not consider horizontal and angular measurements.

Ramirez-Yanes et al^15^ carried out a study to determine the prevalence of mandibular asymmetry, proposing the analysis of digitized panoramic radiographs of 327 children. They found that half of the sample had moderate to severe mandibular asymmetry. The authors used the inter-incisive point as reference to determine the corpus of the mandible. However, prevalence may be overestimated, as a patient may exhibit a dental deviation that alters the point of reference and consequently affects measurement of mandible length.

One difference in the present analysis is the use of points on the maxilla (ANS, RPF and LPF), which is a stable bone and serves as reference for tracing the median sagittal plane. The advantage of this plane is that it corresponds to the true midline, thereby facilitating diagnosis of skeletal (Pg-MSP) and dental (IP-MSP) deviation. With regard to the length of the corpus of the mandible on both sides (LCL, RCL), the reference in the present study was an anatomic point on the mandible (Pg), which is the midpoint of the mandible often seen in orthopantomograms as an white spot on the midline.^14^ Thus, this analysis can also be applied to patients with missing incisors, regardless of the type of malocclusion.

Comparison of measurements revealed considerable discrepancy in the length of mandibular corpus as well as the positioning of the condyles in the patient with posterior crossbite. According to the criteria proposed by Ramirez-Yanes et al,^15^ this suggests significant asymmetry. Analysis of measurements demonstrates that patients (crossbite group) had both skeletal (CL) and positional (CHD) asymmetry. At times, even in cases of obvious mandibular asymmetry, it is not self-evident whether one side has overgrown or the other has undergrown,^24,25^ which underscores the applicability of the analysis proposed herein.

Unlike other analyses available in the literature,^7,8,9,14,15^ this analysis is also based on the visualization of positional asymmetry of condyles through CHD measurement as well as skeletal asymmetry through measurements of the ramus (LRH and RRH), providing a differential diagnosis and assisting in the choice of adequate treatment. It is worth noting that the mandible adapts to mandibular deviations by modeling the condyle and glenoid fossae,^26^ suggesting that asymmetry may be an adaptive response to functional demands.^27^ Animal studies as well as studies involving humans with crossbite have shown that functional shift can produce asymmetric mandibular growth.^7,22^ Children in deciduous and mixed dentition with unilateral posterior crossbite have asymmetrically positioned condyles and asymmetric muscle function. The condyles on the crossbite side are positioned relatively more upwardly and backwardly in the glenoid fossae than the condyles on the non-crossbite side.^29^ Therefore, the prevalence of mandibular asymmetries in young growing patients must be further studied, along with the impact these asymmetries may have on facial growth.^15^

The use of panoramic radiographs to diagnose mandibular asymmetries is subject to distortions, especially in horizontal and oblique measurements;^12,13,17^ thus, a Distortion Factor (DF) is recommended.^15^ It should be calculated for each hemimandible so as to ensure greater accuracy in horizontal measurements (more subject to distortion), and, as a consequence, in diagnosis. This tool (DF) is available in the plug-in. Although there are more effective and accurate diagnostic methods, for instance cone-beam computed tomography (CBCT), panoramic radiography is still widespread,^29^ especially in emerging countries.

## CONCLUSIONS

The analysis proposed herein has the advantage of simultaneously assessing horizontal, vertical and angular mandibular measurements in patients with and without posterior crossbite, thereby allowing differential diagnosis between functional and morphological asymmetry. This easy-to-use public domain tool proves to be an important resource for planning of early orthodontic intervention, in addition to avoiding the progression of asymmetries and their consequences.

## Figures and Tables

**Figure 3:**
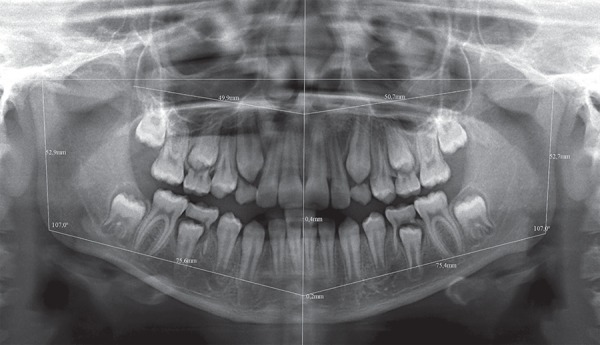
Lemos asymmetry analysis performed on patient with normal occlusion.

**Figure 4:**
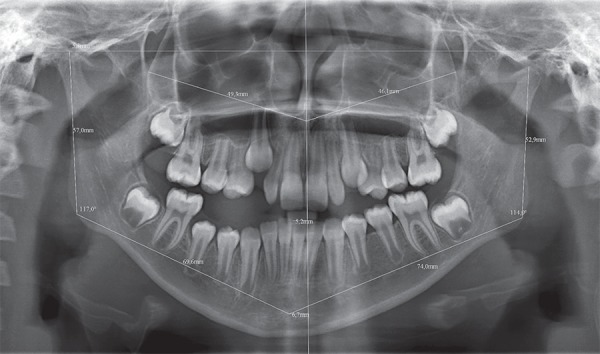
Lemos asymmetry analysis performed on patient with unilateral (right side) posterior crossbite.
